# Temporal Trends of Dengue Surveillance in Sardinia, Italy: Implications of Climate Change on Human and Entomological Monitoring

**DOI:** 10.3390/medicina61112024

**Published:** 2025-11-12

**Authors:** Giovanna Deiana, Isabella Figoni, Antonella Arghittu, Guglielmo Campus, Giuseppe Satta, Cipriano Foxi, Andrea Piana, Paolo Castiglia, Marco Dettori

**Affiliations:** 1 Medical Management, Hygiene, Epidemiology and Hospital Infection, University Hospital of Sassari, 07100 Sassari, Italy; piana@uniss.it (A.P.); paolo.castiglia@uniss.it (P.C.); 2 Public Health Department, Local Health Authority of of Sassari, 07100 Sassari, Italy; isabellafigoni@hotmail.it; 3 Department of Medicine Surgery and Pharmacy, University of Sassari, 07100 Sassari, Italy; aarghittu@uniss.it; 4 Department of Cariology, Institute of Odontology, Sahlgrenska Academy, University of Gothenburg, 413 90 Gothenburg, Sweden; guglielmo.giuseppe.campus@gu.se; 5 Department of Cariology, Saveetha Dental College and Hospitals, Velappanchavadi, Chennai 600 077, India; 6 Department of Oral and Maxillofacial Sciences, Sapienza University of Rome, 00161 Rome, Italy; 7 Experimental Zooprophylactic Institute of Sardinia, 07100 Sassari, Italy; giuseppe.satta@izs-sardegna.it (G.S.); cipriano.foxi@izs-sardegna.it (C.F.)

**Keywords:** *Aedes albopictus*, arbovirus surveillance, Dengue, climate change, vector-borne diseases

## Abstract

*Background and Objectives:* Climate change is modifying the ecological and climatic conditions that influence the distribution and activity of arthropod vectors. Rising temperatures and prolonged warm seasons have favored the establishment of *Aedes albopictus* in Mediterranean regions, increasing the risk of autochthonous Dengue transmission. Therefore, this study describes the evolution of Dengue surveillance in Sardinia between 2018 and 2024, integrating human and entomological data to assess trends, system performance, and implications for prevention and control. *Materials and Methods*: Data on human cases were retrieved from national notification systems (namely PREMAL, arbo.iss.it) and the New Health Information System. Entomological surveillance data were obtained from the Experimental Zooprophylactic Institute of Sardinia. Mosquitoes were collected using BG-Sentinel^®^ traps and ovitraps, covering major cities and points of entry. Descriptive analyses were conducted for both datasets. *Results*: Sixteen Dengue cases were reported during the study period, all imported and laboratory-confirmed in 81% of cases. Most patients were adults (mean age 38 years), and 77% required hospitalization. The most frequent travel origins were Southeast Asia, Africa, and Latin America. No autochthonous cases were identified. Entomological surveillance showed a progressive increase in *Aedes albopictus* captures from 2020 onwards, with seasonal peaks between September and October. Despite intensified sampling and expanded geographic coverage, no mosquito pools tested positive for the Dengue virus. *Conclusions*: Although no locally acquired Dengue infections have been detected, the widespread and increasing presence of *Aedes albopictus* indicates that Sardinia meets the ecological prerequisites for possible autochthonous transmission. Strengthening the timeliness and completeness of human surveillance, improving clinicians’ awareness of reporting requirements, promoting vaccination for travelers, and maintaining continuous entomological monitoring are essential to prevent and promptly manage future outbreaks.

## 1. Introduction

The phenomenon of climate change is profoundly altering the environmental conditions that determine the distribution and activity of arthropod vectors. Increases in average temperature, changes in precipitation patterns, and longer warm seasons could profoundly influence vector physiology, behavior, survival, and biting rate, ultimately affecting vector-borne disease transmission dynamics [1,2,3,4]. These environmental shifts, therefore, play a pivotal role in expanding the geographical range and seasonal window of several vector-borne diseases, as the interaction between vectors, pathogens, and hosts is closely linked to specific climatic conditions [5,6].

Dengue is currently one of the most widespread mosquito-borne viral infections. Transmitted primarily by *Aedes aegypti* and *Aedes albopictus* mosquitoes, it poses a major public health challenge in tropical and subtropical regions [7,8,9,10]. Approximately half of the global population lives in areas at risk, and the World Health Organization estimates that 100–400 million infections occur annually. Reported cases increased from about 500,000 in 2000 to 14.6 million in 2024, although the true burden of disease is likely underestimated due to asymptomatic cases and misdiagnosed infections [11,12,13].

The Americas are the most affected regions with over 13 million cases having been reported in 2024, resulting in more than 7000 deaths [10]. In continental Europe, *Aedes albopictus* has established stable populations in several countries, enabling sporadic local transmission of arboviruses. Autochthonous Dengue cases have been reported in France, Italy, and Spain, demonstrating that suitable ecological conditions can sustain limited outbreaks [14,15,16,17].

In Italy, Dengue is among the arboviral diseases under enhanced surveillance by the National Institute of Health in collaboration with the Ministry of Health, alongside Chikungunya, Zika, West Nile Virus, Usutu virus, Tick-Borne Encephalitis, and Toscana virus neuroinvasive infections. In 2024, approximately 300 locally acquired Dengue cases were reported, mainly in the Veneto and Lazio regions [7,18].

Sardinia, an Italian region and the second-largest island in the Mediterranean Sea, has climatic and ecological characteristics increasingly favorable to *Aedes albopictus* proliferation. According to the Regional Environmental Protection Agency of Sardinia (ARPAS), the island has experienced an average temperature increase of approximately 1.5 °C over the past three decades, accompanied by longer and warmer summer seasons that favor Aedes albopictus survival and activity [19]. Rising average temperatures and prolonged warm periods support vector survival and activity, raising concern for possible autochthonous Dengue transmission, particularly following imported cases [20].

Despite the growing number of surveillance initiatives across Europe, long-term evaluations integrating human and entomological data remain limited. This study provides the first temporal assessment (2018–2024) of Dengue surveillance performance in a Mediterranean island context, analyzing human and entomological data to assess trends, system performance, and the implications of prevention and control strategies for vector-borne disease transmission, particularly highlighting the implications of climate change for vector monitoring and public health preparedness.

## 2. Materials and Methods

### 2.1. Human and Entomological Surveillance System

The surveillance of arbovirus in Italy is integrated into the National Plan for the Prevention, Surveillance and Response to Arboviral Diseases 2020–2025, which aims to prevent and mitigate the risk of autochthonous transmission of vector-borne viruses within the national territory. The plan integrates human and entomological surveillance activities to ensure early detection and timely response to potential outbreaks [21].

Human surveillance is continuous throughout the year but is intensified during the period of peak vector activity to ensure timely identification of imported and locally acquired cases [21]. Once laboratory confirmation is obtained, probable and confirmed cases must be immediately reported by Local Health Authorities (LHAs) through the national infectious disease notification system (namely PREMAL) and the National Institute of Health’s dedicated platform for arboviral surveillance [22,23].

Entomological surveillance focuses on identifying mosquito species capable of transmitting arboviruses and detecting the presence of viral pathogens within collected specimens, thereby defining periods of higher transmission risk. These activities are coordinated by the Experimental Zooprophylactic Institutes (EZS), in collaboration with the Veterinary Services of the Regional Health Protection Agencies and local authorities. The EZS also performs laboratory diagnostics and manages the collection and analysis of epidemiological data [21].

### 2.2. Study Setting

The present study describes the temporal trends of Dengue surveillance between 2018 and 2024 in the Sardinia region, covering an area of approximately 24,000 km^2^ with a population of about 1.6 million inhabitants. The island is characterized by a Mediterranean climate, with mild, wet winters and hot, dry summers, creating favorable conditions for the establishment and proliferation of *Aedes albopictus*, particularly from late spring to early autumn.

The regional arbovirus surveillance system is implemented within the framework of the National Plan for the Prevention, Surveillance and Response to Arboviral Diseases 2020–2025, through the collaboration of the EZS of Sardinia, the LHAs, and the Regional Health Protection Agency [21].

### 2.3. Trap Distribution

**Figure 1:**
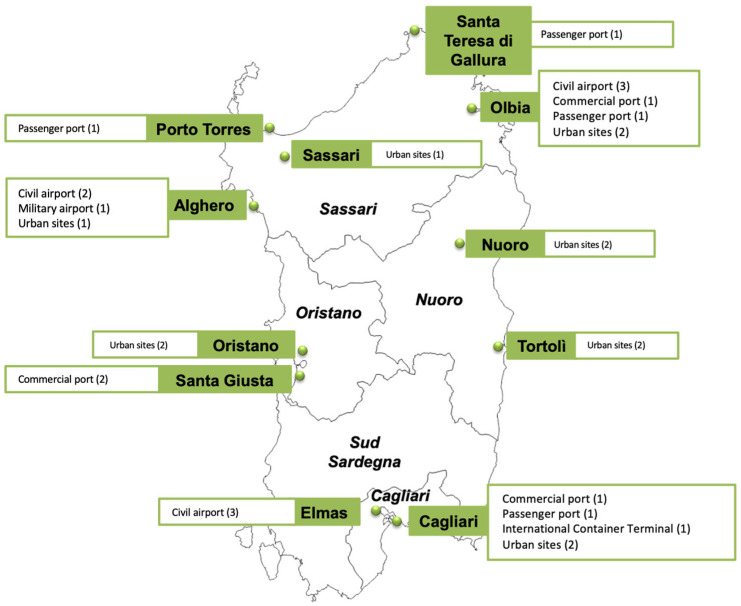
Trap distribution covering the main points of entry and the island’s major cities. Sardinia region, Italy, 2020–2024.

Site selection for trap placement within each area was based on environmental, ecological, and epidemiological parameters, particularly the presence of large stagnant water bodies that support mosquito preimaginal stages, such as ponds, lagoons, and rice fields, as well as altitude. Mosquito captures were conducted every fifteen days from April to October, while in the remaining months, one capture per month was carried out. The targeted surveillance covered the main points of entry (commercial and passenger ports, civilian and military airports) as well as the island’s major cities (Figure 1).

The traps used were BG-Sentinel^®^ (Biogents’ mosquito traps), specifically developed for monitoring mosquitoes of the genus *Aedes*, although they are also capable of capturing other mosquito species. These traps, baited with a specific chemical attractant, were placed outdoors in sheltered areas protected from rain, wind, and direct sunlight, and near larval development sites. The traps were located far away from other strong sources of attraction, to avoid competition or amplification effects. Therefore, proximity to sources of light, heat, carbon dioxide, or other attractants was avoided. The placement also considered the safety of the operator, the trap itself, and the public safety.

Surveillance targeted not only adult specimens but also included the study of eggs using ovitraps (water-filled cups that attract *Aedes* mosquitoes for oviposition). Larval collection was used to enhance the monitoring of mosquito species in each area and served as a valuable tool for assessing the effectiveness of vector control and management interventions.

### 2.4. Sample Handling

Traps were retrieved after sunrise (around 8:00 a.m.), but not too late, to prevent the mosquitoes from dying of dehydration before retrieval and to avoid battery drainage, which would cause the loss of specimens no longer retained by the airflow generated by the fan.

The retrieval process was conducted in a defined sequence. In order to ensure the continued functionality of the fan, mosquitoes were directed towards the base of the collection net. The net or collection chamber was then securely closed using the appropriate tie, taking care not to damage the specimens. Subsequently, the trap was disconnected from its power source and support structure, and all materials and equipment were carefully collected.

During transport, the bags and containers were kept refrigerated (+4 °C) to prevent the insects from being crushed. The insects were killed by placing the nets in a freezer at −20 °C for at least 15–30 min.

Afterwards, the killed insects were placed in Falcon-type tubes between two layers of loosely packed cotton wool. The tube contained about one-fourth of its volume of silica gel or another desiccant to prevent mold formation. The two cotton layers were placed in such a way that the samples do not move during transport, which could have damaged key features needed for identification, but without compressing the specimens.

Each sample was labelled (trap type, location, and date) and accompanied by a specific form. One form was used for each collection date. The collection date referred to the morning on which the insects were retrieved.

### 2.5. Data Sources and Analysis

Data on human surveillance were obtained from the New Health Information System and from the National Institute of Health arboviral surveillance platform. The national platform provided access to reported cases from 2020 onwards, whereas for the years before 2020, all Dengue reporting forms submitted by the LHAs were retrieved directly from PREMAL [21].

Both the PREMAL system and the arboviral surveillance platform operate within the standardized national infectious disease surveillance framework coordinated by the Italian National Institute of Health, which includes routine validation and quality assurance procedures to ensure data completeness and reliability.

Entomological surveillance data were derived from reports produced by the EZS of Sardinia. For 2018–2019, data were extracted from the ISTISAN (Istituto Superiore di Sanità—National Institute of Health) Reports 22/22, while for subsequent years, they were obtained directly from EZS regional documentation [24].

All data were compiled, organized, and analyzed using Microsoft Excel (Microsoft Office, Redmond, WA, USA).

## 3. Results

### 3.1. Human Surveillance

A total of 16 cases of Dengue virus infection were reported during the study period. All were classified as imported cases, as the patients had travelled outside Italy within 15 days before symptom onset. Of these, 13 (81.25%) were confirmed by the regional reference laboratory of Cagliari, through the detection of a specific antibody response or by viral isolation. The majority of notifications originated from the LHA of Cagliari (8 cases), followed by Sassari (3 cases) and Gallura (1 case). In four instances, the reporting LHA could not be identified due to incomplete notification forms.

Regarding confirmed cases, the distribution by gender showed a higher prevalence among females (8 females vs. 5 males). Patients ranged in age from 10 to 84 years (mean 38 years), with most cases involving individuals aged between 20 and 50 years.

From a clinical perspective, all cases exhibited fever. The most frequently reported symptoms included general malaise, fatigue, headache, and joint pain. A minority of patients also exhibited gastrointestinal symptoms such as nausea, vomiting, and diarrhea, as well as cutaneous rash. Approximately 20% presented with hematological abnormalities, specifically leukopenia and thrombocytopenia; only one of these cases progressed to hemorrhagic manifestations. Retro-orbital pain was reported in less than 20% of cases.

Ten of the thirteen confirmed cases (76.92%) required hospitalization. A single case of re-infection was identified, associated with a higher risk of complications; however, all cases had a favorable clinical outcome, with complete recovery or significant improvement.

**Figure 2:**
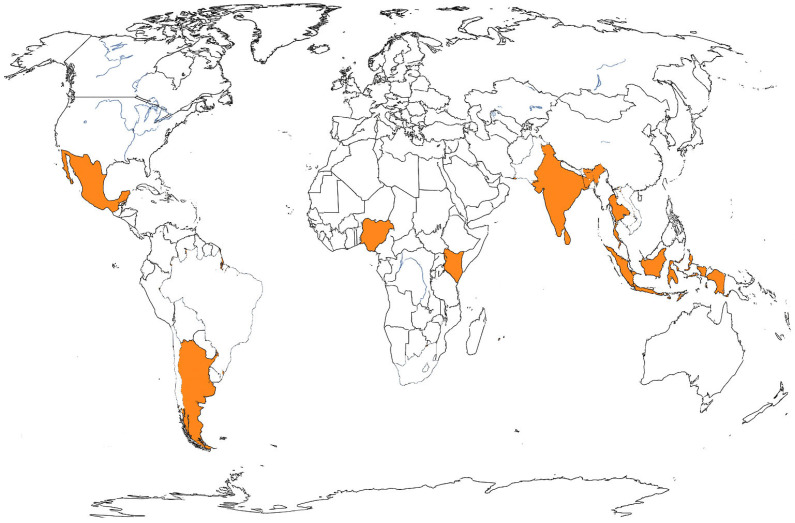
Countries in orange indicate the origins of imported Dengue cases. Sardinia region, Italy, 2020–2024.

In consideration of the fact that all cases were imported, the documentation of the respective countries of origin was also undertaken. The following countries were represented: Thailand (4 cases), the Maldives (2), Cameroon (2), India, Dominican Republic, Kenya, Guatemala, Barbados, Argentina, Indonesia, and Mexico (Figure 2).

The times of case reporting could be assessed in only 7 instances where the date of initial notification by the physician to the LHA was available. In these cases, the LHA complied with the reporting deadlines set by the National Arbovirus Surveillance Plan, transmitting the information to the Ministry of Health and the National Institute of Health. In 2 cases, delays of 1 and 8 days, respectively, were recorded.

### 3.2. Entomological Surveillance

In 2018, entomological monitoring was carried out in 36 locations using 38 traps, resulting in 665 sampling events and the capture of 13,128 mosquitoes. In 2019, sampling was performed with 44 traps distributed across 42 different locations (Table 1).

Due to logistical constraints, specifically the lack of dry ice, CDC-light traps were deployed without CO_2_, reducing capture efficiency. A total of 606 captures were carried out, yielding 2663 culicids across 14 species. The most abundant species were *Culex pipiens* (47%) and *Ochlerotatus caspius* (17%). This latter species has been recently included within the *Aedes* genus.

All captured specimens were transported to the laboratory for morphological identification and then grouped into pools (up to 25 mosquitos per pool) based on species, capture date, and location. These pools were submitted to the Virology Laboratory for pathogen screening, including Dengue virus. To date, no pools have tested positive. Moreover, *Aedes albopictus* was rarely collected during the 2018–2019 campaigns, likely due to the use of trap types and rural sampling sites that are suboptimal for detecting this diurnal, predominantly urban species.

Nonetheless, the entomological data collected contributed to the characterization of species distribution, abundance, and potential vector presence. Notably, in 2018, a higher number of captures was observed, likely attributable to the stronger attractant effect of CO_2_, which was not used in 2019.

**Figure 3:**
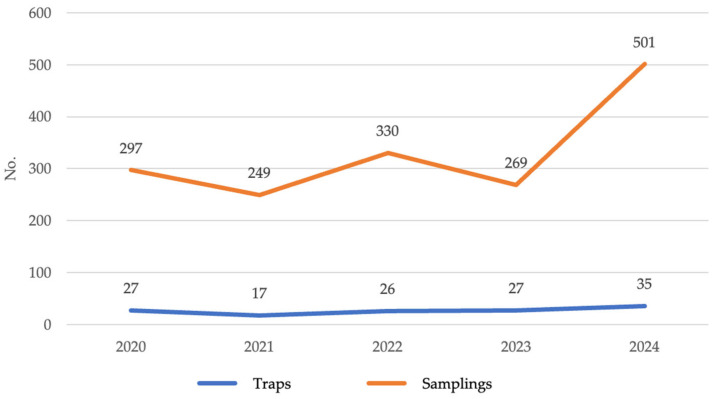
Temporal variations in trapping sites and sampling frequency. Sardinia region, Italy, 2020–2024.

From 2020 to 2024, surveillance data were derived exclusively from BG-Sentinel traps, which are optimized for capturing *Aedes* mosquitoes and other diurnal species but are less effective for culicids. The annual sampling efforts are summarized in Figure 3.

The data show a progressive increase in sampling activity, with 1167, 980, 2395, 3272, and 2283 mosquitoes captured over the study years, reflecting two main factors. Firstly, there was an expansion in the number of traps, particularly in 2024. Secondly, there was a greater frequency of trapping, resulting in wider geographic surveillance coverage.

**Figure 4:**
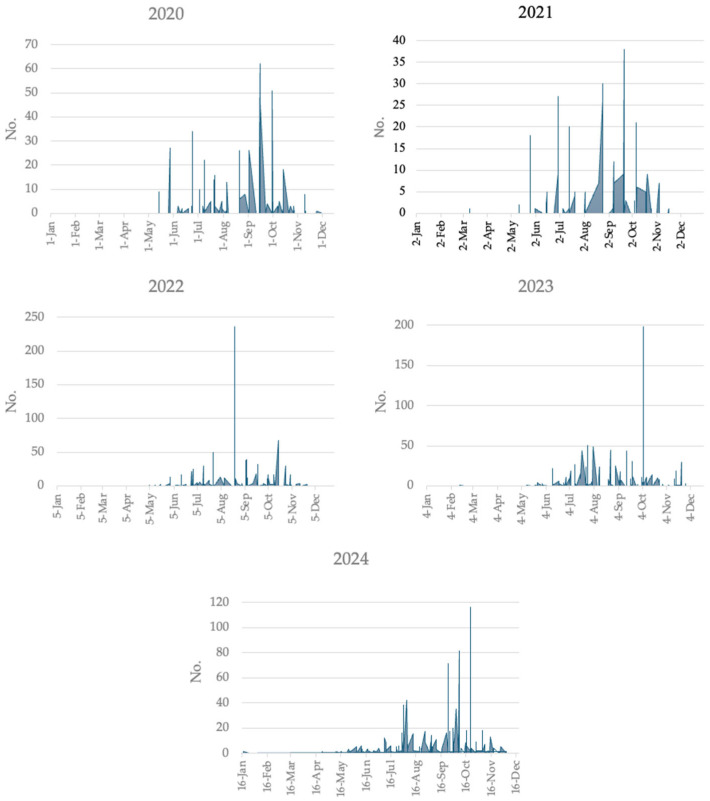
Temporal distribution of *Aedes albopictus* captures. Sardinia region, Italy, 2020–2024.

The seasonal distribution of *Aedes albopictus* consistently spanned from June to November over the five-year period, with peak abundance typically occurring in October, except in 2022, when a peak was observed in early September (Figure 4).

**Figure 5:**
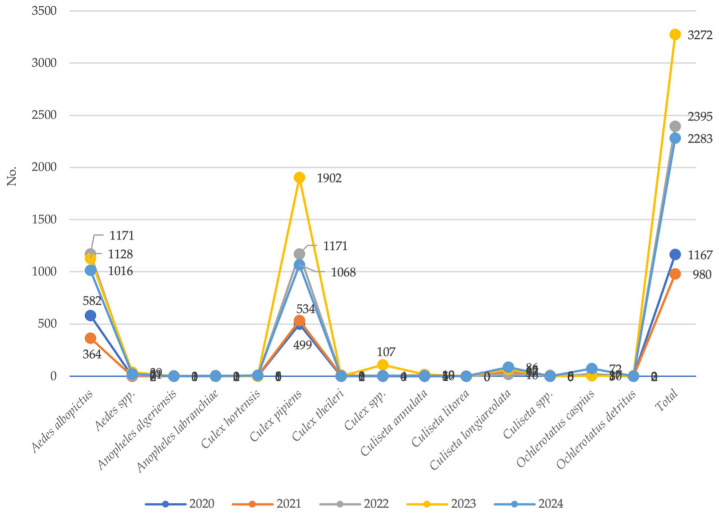
Mosquito species collected using BG traps. Sardinia region, Italy, 2020–2024.

Species captured with BG traps exhibited two main abundance peaks: one for *Aedes albopictus* and one for *Culex pipiens*. Despite being less attracted to this trap type, *Culex pipiens* remained the most frequently detected species overall (Figure 5).

**Figure 6:**
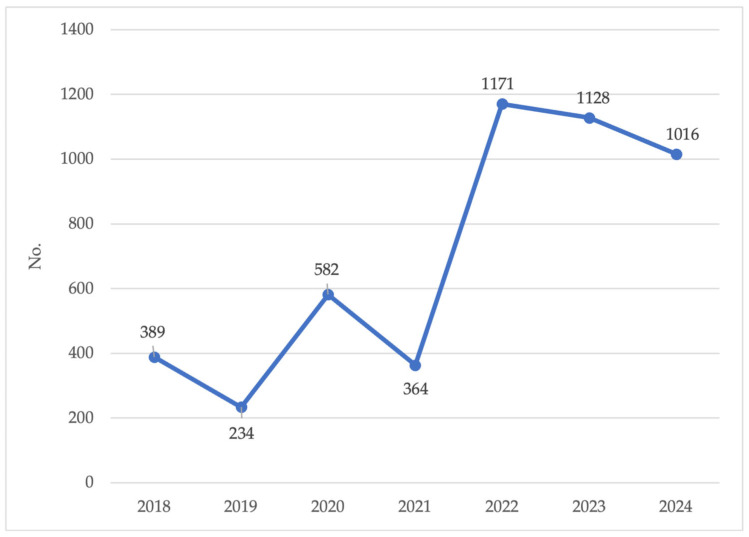
*Aedes albopictus* specimens captured during the period 2018–2024. Sardinia region, Italy.

Focusing on *Aedes albopictus*, the primary vector of concern for Dengue transmission, a marked increase in the total number of captured specimens was observed in the most recent three-year period (2022–2024) compared to 2018–2021, mainly reflecting the expansion of surveillance activities and the increased trapping effort (Figure 6).

A sample of *Aedes albopictus* specimens collected with BG-traps was selected for virological testing. As of January 2025, no samples have tested positive for Dengue virus.

The progressive increase in *Aedes albopictus* captures over time seems to reflect both the expansion of surveillance coverage and a true rise in vector abundance driven by increasingly favorable climatic and ecological conditions.

Together, these findings provide an overview of Dengue surveillance performance and vector trends in Sardinia.

## 4. Discussion

In recent decades, Dengue has emerged as a growing public health threat in Europe. Otherwise, Italy has never been considered an endemic area, a number of environmental and climatic factors are creating favorable conditions for its local transmission [18]. The average temperatures are rising and the expansion of *Aedes albopictus*, a species now widespread throughout the country, are enabling the vector’s survival even during the winter months, thus increasing the risk of autochthonous transmission [25,26].

Recently, autochthonous cases of Dengue have been documented in several Italian regions, demonstrating that local virus transmission is now a reality and not just a possibility [27,28,29]. The continued arrival of infected travelers from endemic areas and the presence of a competent vector are key elements facilitating the emergence of new epidemic outbreaks. European and national epidemiological data highlight the need to closely monitor areas where vectors are present to prevent and manage potential outbreaks of locally acquired Dengue. Surveillance, along with entomological control strategies and public information campaigns, is essential to reduce the risk of local transmission.

The presence of *Aedes albopictus* is also confirmed in Sardinia, where now it is considered an endemic species, well established across the island and reported almost year-round. Moreover, data from EZS monitoring indicate a numerical increase in recent years and a distribution that is mostly homogeneous throughout the region. Its presence is mostly recorded during the summer months, with peaks in late summer. However, available data do not allow us to determine with certainty whether the increase in specimens is due to enhanced surveillance or to an actual increase in mosquito populations.

Monitoring activities are steadily improving in terms of the number of locations covered. The number of trap sites is increasing, as is the number of samples collected.

Although *Aedes albopictus* is not the primary vector of Dengue, it is fully capable of replicating and transmitting the virus. Therefore, even though no local viral circulation has yet been detected, all the conditions are present for this to occur in the near future [30,31]. The environmental temperatures in Sardinia, particularly during the summer, are also favorable for optimal vector development [24]. This is essential to trigger the biochemical reactions required for viral replication.

Regarding human surveillance, case analysis was conducted using notification forms submitted through two platforms: the National Arbovirus Surveillance System and the new National Health Information System, via the PREMAL application. The forms from both platforms are almost identical in terms of the data collected; differences lie mainly in the mandatory nature of some data fields, which results in slight discrepancies in the information obtained.

From a clinical perspective, most of the cases considered in the study required hospitalization (about 77%), suggesting a severity of symptoms that did not allow for outpatient management, even though Dengue, in cases of primary infection, often presents as asymptomatic or mildly symptomatic [12,32]. This points to a problem of underdiagnosis, which may be related to various factors, such as the nature of the infection itself as mild symptoms may not raise concern in the patient, who may unknowingly become a potential source of virus transmission to mosquitoes. Furthermore, underdiagnosis results in the absence of mosquito control interventions, which may allow the virus to spread, thereby posing risks to the general population [33].

Since most cases of Dengue reported in Italy and all those observed in Sardinia are imported, vaccination can be an important preventive tool for travelers visiting endemic regions. Immunization can significantly reduce the individual risk of infection and, consequently, the likelihood of introducing the virus into areas where competent vectors such as *Aedes albopictus* are established. However, vaccination coverage among travelers remains limited, partly due to the novelty of the vaccine, restricted availability, and limited awareness among both healthcare professionals and the public [34].

Improving medical training is essential to ensure early case recognition. This imperative is further underscored by the need to fortify and streamline the health surveillance system. Moreover, it is crucial to augment healthcare professionals’ cognizance of the paramount importance of prompt case notification. Some notification forms had a delay in reporting beyond the 12 h time frame recommended by the National Arbovirus Plan, highlighting the need for improved education of reporting healthcare professionals regarding the accurate and comprehensive completion of notification forms and the importance of timely reporting. In light of the frequent incompleteness of essential fields, it is recommended that the inclusion of key data be made mandatory. Such data should include laboratory results, the date of notification from the attending physician to the Local Health Authority, the reporting healthcare facility, the case’s place of residence (a crucial element in the event of outbreak development), and the return date to Italy in imported cases.

## 5. Conclusions

Despite the absence of autochthonous Dengue cases in Sardinia at the present time, this study documents the presence of all the necessary ecological and epidemiological preconditions for local transmission. In order to prevent future outbreaks and provide support, it is essential to improve the sensitivity of human surveillance, ensure complete and timely reporting, promote vaccination where indicated, and increase measures to control the spread of the vector.

Future efforts should focus on integrating climatic and environmental data with predictive modeling approaches to enhance Dengue surveillance in Mediterranean settings. Combining entomological, epidemiological, and meteorological information may allow the development of early warning systems capable of anticipating periods and areas at higher risk of transmission. Such models could support targeted interventions, optimize resource allocation, and strengthen preparedness strategies in regions like Sardinia, where ecological conditions are increasingly favorable for vector-borne disease circulation.

## Figures and Tables

**Table 1:** Mosquito species captured during 2018 and 2019. Sardinia region, Italy.

Species	2018			2019		
	M	F	Total	M	F	Total
*Aedes albopictus*	85	304	389	78	156	234
*Aedes* spp.	2	81	83	7	68	75
*Aedes vexans*	0	192	192	0	8	8
*Anopheles algeriensis*	0	93	93	1	15	16
*Anopheles labranchiae*	3	200	203	1	56	57
*Anopheles* spp.	0	3	3	0	6	6
*Coquillettidia buxtoni*	0	1	1	0	7	7
*Coquillettidia richiardii*	0	1	1	0	0	0
*Culex hortensis*	2	7	9	10	21	31
*Culex mimeticus*	0	1	1	0	0	0
*Culex pipiens*	214	3818	4032	230	1034	1264
*Culex* spp.	12	78	90	24	47	71
*Culex theileri*	13	988	1001	0	62	62
*Culiseta annulata*	39	1019	1058	6	160	166
*Culiseta longiareolata*	50	95	145	39	36	75
*Culiseta* spp.	1	10	11	1	11	12
*Culiseta subochrea*	0	9	9	0	2	2
*Ochlerotatus berlandi*	0	2	2	0	0	0
*Ochlerotatus caspius*	64	4683	4747	12	442	454
*Ochlerotatus detritus*	35	984	1019	2	116	118
*Ochlerotatus mariae*	0	0	0	0	5	5
*Uranotaenia unguiculata*	0	39	39	0	0	0
Total	520	12,608	13,128	411	2252	2663

## Data Availability

Data are available on reasonable request.

## Abbreviations

The following abbreviations are used in this manuscript:

LHA	Local Health Authorities
EZS	Experimental Zooprophylactic Institutes
